# Outcomes of Total Joint Arthroplasty in Black, Asian, Minority Ethnic Groups Versus Local Population: A Retrospective Review

**DOI:** 10.7759/cureus.19868

**Published:** 2021-11-24

**Authors:** Mohammad Noah Khan, Muhammad U Ali, Lokesh Bhambani, Nagraj Prashanth, Samantha Tross

**Affiliations:** 1 Trauma and Orthopaedics, Royal Victoria Hospital, Belfast, GBR; 2 Trauma and Orthopaedics, Health Education Northwest London, London, GBR; 3 Trauma and Orthopaedics, London Northwest NHS Trust, London, GBR; 4 Trauma and Orthopaedics, London Northwest Trust, London, GBR

**Keywords:** oxford score, complications, bame, hip arthroplasty, knee arthroplasty

## Abstract

Background: Total hip and knee replacement decrease the disability caused by osteoarthritis of the lower extremities. Although it has been established that racial and ethnic minorities underutilize these procedures, little data on postoperative outcomes exists. The impact of race on postoperative Oxford scores and complications following total joint arthroplasty (TJA) will be investigated in this retrospective review.

Methods: A retrospective review of 120 elective primary TJA procedures was undertaken between January 2016 and December 2019 in a single institution. To measure variations between the various groups, t-tests were used on their Oxford scores, and chi-squared bivariate regression was used to classify all categorical variables and the association of ethnicity and surgery type with gender.

Results: There were 62 (51.6%) White patients and 59 (49.1.0%) Black, Asian, Minority Ethnic (BAME) patients in total. The majority of the patients were females (60.9% vs 39.2%, p = 0.032). Low vitamin D levels were seen in a small percentage of patients in the sample (15.8% vs 84.2%, p = 0.460). There is a statistically important connection (p = 0.001) between the surgery type (total knee replacement [TKR]/total hip replacement [THR]) and gender; 41 females had TKR surgery, and 32 had THR surgery.

Conclusion: The study found that the relationships between ethnicity (White/BAME) and gender as well as surgery type (TKR/THR) and gender are statistically important. In all cases with low vitamin D and normal vitamin D levels, White patients had higher overall Oxford hip scores than the BAME patients. To comprehend the differences discovered, further research is needed. To try to eliminate the difference, targeted approaches should be created.

## Introduction

Minority groups, including racial and ethnic minorities, encounter disparities in access, use, and outcomes in the healthcare system. Minorities in the United Kingdom already make up more than 13% of the overall population with the Asian community being the majority at 7.5% [1]. It is not enough to simply understand where these inequalities exist; as healthcare providers, we have an ethical duty to discuss and eradicate them. The prevalence of inequalities in health does not only concern vulnerable groups, we all also suffer from them [2].

The majority of patients report improvements in pain, quality of life, and physical and mental well-being after total joint arthroplasty (TJA), and the rate of short-term complications recorded in the literature for patients undergoing TJA is minimal [3-8]. Despite this, there are persistent differences in TJA use rates between White and Black, Asian, Minority Ethnic (BAME) patients. According to research, BAME patients are less likely than White patients of equal disease severity to undergo TJA [9]. Several factors are hypothesized as contributing to racial disparities including lower health literacy, differences in socioeconomic status, geographical location, and decreased trust in the medical field though none of these conclusively explain the differences [10-15]. Identifying which factors affect specific outcomes can aid in the development of tailored interventions to address the existing inequalities.

Infection and arthrofibrosis are two short-term complications of TJA that have a well-defined range of patient risk factors, including elevated BMI, diabetes mellitus (DM), smoking, and an increased American Society of Anesthesiologists (ASA) score [16-19]. Although patient variables are less linked to periprosthetic fracture and early dislocation, several studies have linked female sex, age, and BMI to both of these complications [20-24].

With this research, we aim to compare the results of BAME and non-BAME patients after TJA in a single high-volume hospital as well as examine the impact of race on short-term complications after. We hypothesize that race and vitamin D levels are important factors in variation in postoperative outcomes.

## Materials and methods

The research was registered with the hospital's clinical governance department as a quality improvement project. Following registration, all cumulative joint arthroplasties (TJA) performed in the center between January 2016 and December 2019 were checked retrospectively. Age, gender, race, procedure, pre-operative vitamin D levels, and Oxford hip scores at six weeks, three months, six months, and one year were all variables in the sample. All primary joint replacements on the lower limbs, such as total hip arthroplasty (THA) and total knee arthroplasty (TKA), were included in the study. Both upper limb joint replacements and all revision joint replacements were excluded from the study.

Patients who required joint arthroplasties in an acute environment, such as after a traumatic injury, were excluded. Patients not having complete records for the variables being investigated were excluded from the study. Patient records and clinic letters were examined, and a year of postoperative follow-up was established as the standard for inclusion. Patients who had been followed for less than a year were removed from the study. All the patients included in our review were operated on by the same surgeon to reduce bias such as approach, prosthesis choice, capsule repair, and closure. There were a total of 120 patients chosen. Oxford scores were used to monitor postoperative outcomes. The chi-square test was used to determine the significance of variables such as ethnicity and surgery type with gender. Independent-samples t-test was used for comparison of White vs BAME patients with low vitamin D levels, and analysis of Oxford hip scores at six weeks, three months, six months, and one year was done.

## Results

All categorical variables were analyzed using the chi-square test, and there was a significant association of ethnicity and surgery type with gender. Out of 120 patients, 47 (39.2%) were males and 73 (60.9%) were females (Table 1); 48.4% (58) of the patients were of the BAME population, and 51.7% (62) of the patients were of White descent. Of the females, 41 were of the BAME group and 32 were White. Seventeen males were of the BAME group and 30 were White (Table 1). An association between ethnicity (White/BAME) and gender is statistically significant (p = 0.03); 41.7% of the studied patients had a total knee replacement (TKR) with the majority (34.2%) being women (Table 1). A total of 70 (58.4%) patients had a total hip replacement (THR), out of which 32 were females. Only nine males had TKR surgery, but 38 had gone through THR surgery.

**Table 1:** Chi-square test to show the association between different variables VitD: Vitamin D; BAME: Black, Asian, Minority Ethnic; TKR: total knee replacement; THR: total hip replacement.

Variables	Gender		
	Male	Female	P-value
Ethnicity			
White	30 (25)	32 (26.7)	0.032
BAME	17 (14.2)	41 (34.2)	
VitD levels			
Low	6 (5)	13 (10.8)	0.460
Normal	41 (34.2)	60 (50)	
Surgery types			
TKR	9 (7.5)	41 (34.2)	0.001
THR	38 (31.7)	32 (26.7)	
Ethnicity	Low VitD	Normal VitD	
White	10 (8.3)	52 (43.3)	0.927
BAME	9 (7.5)	49 (40.8)	

Association between surgery type (TKR/THR) and gender was seen to be statistically significant (p = 0.001). The majority of the patients in the study (84.2%) had normal vitamin D levels, and a small percentage (15.8%) had a low vitamin D level. Ten (8.3%) patients with low vitamin D levels were of the White population, whereas nine (7.5%) patients were of the BAME group (Table 1). Gender and ethnicity have shown no association with vitamin D levels (p = 0.927).

An independent-samples t-test was carried out for low vitamin D levels in White/BAME patients with Oxford hip scores taken at six weeks, three months, six months, and one year. The same test was followed for patients with normal vitamin D levels. Our study showed that nine BAME group patients and 10 White patients from the study had low vitamin D levels, whereas 52 White patients and 49 BAME patients had a normal vitamin D level (Table 1). For low vitamin D group, there was no significant change in the Oxford score between BAME and non-BAME population at six weeks (p = 0.149), three months (p = 0.696), six months (p = 0.236), or one year (p = 0.210). The same was the case with the normal vitamin D group even after one year (p = 0.288). In both cases of low vitamin D and normal vitamin D levels, this study found that White participants had higher average Oxford hip scores than the BAME patients by using an independent-samples t-test. We concluded from Levene's test that the variation in Oxford hip scores in White patients is not statistically significantly different from that in BAME patients (Tables 2, 3).

**Table 2:** Independent-samples t-test for low VitD level in White/BAME patients with Oxford hip scores (6 weeks, 3 months, 6 months, and 1 year) VitD: Vitamin D; BAME: Black, Asian, Minority Ethnic.

			N	Mean	Std	F-stats	P-value
VitD Low	6 Weeks	White	10	29.90	6.027	2.283	0.149
		BAME	9	21.89	2.667		
	3 Months	White	10	37.40	6.687	0.158	0.696
		BAME	9	28.89	6.735		
	6 Months	White	10	41.50	3.536	1.510	0.236
		BAME	9	33.56	6.002		
	1 Year	White	10	44.90	1.969	1.699	0.210
		BAME	9	38.44	3.941		

**Table 3:** Normal VitD level in White/BAME patients with Oxford hip scores (6 weeks, 3 months, 6 months, and 1 year) VitD: Vitamin D; BAME: Black, Asian, Minority Ethnic.

			N	Mean	Std	F-stats	P-value
VitD Normal	6 Weeks	White	52	29.19	6.756	3.749	0.056
		BAME	49	22.24	8.705		
	3 Months	White	52	32.69	8.048	2.148	0.146
		BAME	49	27.24	9.470		
	6 Months	White	52	35.44	9.898	0.113	0.738
		BAME	49	31.12	9.048		
	1 Year	White	52	38.90	7.354	1.140	0.288

## Discussion

Although several types of research have been conducted to investigate the field of inequalities in healthcare delivery, disparities continue to be a major concern and tend to exist regardless of social status, insurance status, clinical appropriateness, or treatment location [25]. With the growing number of total hip and TKA procedures performed each year in the United Kingdom, it is critical that the treatment we provide is of high quality, reliable, and cost-effective for both the patients and the overall health system. However, it is also critical that we do not enable our attempts to improve productivity and cost-effectiveness to be used to reinforce continuing healthcare inequity. It is important to identify places where inequalities occur to enhance the treatment and outcomes. For TJA patients, race is an environment where inequalities are known to occur, and these disparities have persisted over time [26]. Short-term complications after TJA are also a major contributor to higher healthcare costs and lower medical outcomes [27]. While it is unlikely that we will be able to fully eradicate the short-term postoperative complications following TJA, even a small reduction in these complications may result in substantial cost savings as well as improved patient satisfaction and outcomes. At the same time, providers have an ethical obligation to ensure that accounting for these risk factors does not exacerbate the existing inequalities [28].

As reported in the findings, there were significant differences between White and BAME patients, but certain factors, such as BMI and health status, were not taken into account. Excluded causes may have a negative impact on medical outcomes, and the majority of them are potentially modifiable. BMI has been linked to postoperative complications such as inflammation, reduced prosthesis survival, and worse functional outcomes [29-33]. BMI is also the simplest risk factor to change, with even small changes boosting perioperative and postoperative risks [34]. In terms of co-morbidities that can affect the outcomes, diabetes is worrying, given the comprehensive literature linking diabetes to elevated complication rates in TJA patients [35-38]. Racial inequalities are multifactorial, with patient, provider, and device variables as contributing factors [39]. Many of these contributing factors are possibly modifiable, but further research is required to pinpoint them. Examining the experiences of various variables, such as the patient’s treatment from the provider, care team, and organization, as well as factors outside of the healthcare system, such as social support, is vital to eradicating current inequalities [40]. Patient results are influenced by social determinants of health, which can have an even greater effect than medical treatments [41,42]. In order to better understand and eventually eradicate the current inequalities, the patient, provider, and system variables at various levels, such as provider, care staff, organizational, and environmental levels, must be understood.

This research has a range of advantages. We were able to collect a broad sample from a single high-volume hospital, and all patients were subjected to the same perioperative procedures, thus reducing the number of confounding variables. We were also able to obtain more accurate patient details since it was a single institution report. Our sample’s ethnic makeup strongly resembles that of our immediate surroundings. Our population is still somewhat homogeneous in terms of social class, which is a strength since it not only reduces the confounding variables but also restricts the applicability of a broader variety of organizations. Since the findings may not apply to all practice settings, the single institution is also a constraint. Due to the retrospective aspect of the research, there are also inherent limitations.

## Conclusions

Although several studies have revealed evidence of health inequities in healthcare, we found no differences in the TKR/THR results in terms of outcome scores and vitamin D levels. Our study discovered that there is a statistically significant relationship between ethnicity (White/BAME) and gender as well as surgery form (TKR/THR) and gender. We found that women are more likely to undergo joint replacements than men, and Whites have better outcomes compared to BAME based on their average Oxford hip scores in all cases with low vitamin D and normal vitamin D levels. More research is required to fully comprehend the differences discovered in this analysis. Targeted methods should be developed to try to eliminate the disparity.
